# Development of a Fluorescent Assay to Search New Drugs Using Stable tdTomato-*Leishmania*, and the Selection of Galangin as a Candidate With Anti-Leishmanial Activity

**DOI:** 10.3389/fcimb.2021.666746

**Published:** 2021-06-04

**Authors:** María Fernanda García-Bustos, Agustín Moya Álvarez, Cecilia Pérez Brandan, Cecilia Parodi, Andrea Mabel Sosa, Valeria Carolina Buttazzoni Zuñiga, Oscar Marcelo Pastrana, Paula Manghera, Pablo Alejandro Peñalva, Jorge Diego Marco, Paola Andrea Barroso

**Affiliations:** ^1^ Escuela Universitaria en Ciencias de la Salud y Facultad de Ciencias Agrarias y Veterinarias, Universidad Católica de Salta, Salta, Argentina; ^2^ Instituto de Patología Experimental, Consejo Nacional de Investigaciones Científicas y Técnicas (CONICET) - Universidad Nacional de Salta, Salta, Argentina; ^3^ Facultad de Ciencias de la Salud, Universidad Nacional de Salta, Salta, Argentina

**Keywords:** tdTomato, *L. (L.) amazonensis*, flavonoids, galangin, fluorescence

## Abstract

Antimonials continue to be considered the first-line treatment for leishmaniases, but its use entails a wide range of side effects and serious reactions. The search of new drugs requires the development of methods more sensitive and faster than the conventional ones. We developed and validated a fluorescence assay based in the expression of tdTomato protein by *Leishmania*, and we applied this method to evaluate the activity *in vitro* of flavonoids and reference drugs. The pIR1SAT/tdTomato was constructed and integrated into the genome of *Leishmania (Leishmania) amazonensis.* Parasites were selected with nourseothricin (NTC). The relation of *L. amaz*/tc3 fluorescence and the number of parasites was determined; then the growth *in vitro* and infectivity in BALB/c mice was characterized. To validate the fluorescence assay, the efficacy of miltefosine and meglumine antimoniate was compared with the conventional methods. After that, the method was used to assess *in vitro* the activity of flavonoids; and the mechanism of action of the most active compound was evaluated by transmission electron microscopy and ELISA. A linear correlation was observed between the emission of fluorescence of *L. amaz*/tc3 and the number of parasites (r^2^ = 0.98), and the fluorescence was stable in the absence of NTC. No differences were observed in terms of infectivity between *L. amaz*/tc3 and wild strain. The efficacy of miltefosine and meglumine antimoniate determined by the fluorescence assay and the microscopic test showed no differences, however, *in vivo* the fluorescence assay was more sensitive than limiting dilution assay. Screening assay revealed that the flavonoid galangin (GAL) was the most active compound with IC_50_ values of 53.09 µM and 20.59 µM in promastigotes and intracellular amastigotes, respectively. Furthermore, GAL induced mitochondrial swelling, lipid inclusion bodies and vacuolization in promastigotes; and up-modulated the production of IL-12 p70 in infected macrophages. The fluorescence assay is a useful tool to assess the anti-leishmanial activity of new compounds. However, the assay has some limitations in the macrophage-amastigote model that might be related with an interfere of flavanol aglycones with the fluorescence readout of tdTomato. Finally, GAL is a promising candidate for the development of new treatment against the leishmaniasis.

## Introduction

The leishmaniases are a group of diseases caused by parasites of the genus *Leishmania*, which are transmitted to humans through the bite of infected female phlebotomine sandflies. These diseases in all their clinical expressions are endemic in 98 countries (Alvar et al., 2012), affecting mainly low income populations of the tropical and subtropical belt worldwide. According to the WHO report (2016), over one billion people live in endemic areas at risk of infection (World Health Organization, 2016), and an estimated 900.000 – 1.6 million new cases and 20.000 to 40.000 deaths occur annually (Alvar et al., 2012).

Depending on the infecting *Leishmania* species and the immune response of the host, leishmaniases can exhibit a disease spectrum that includes visceral leishmaniasis or “kala-azar”, affecting mainly the mononuclear phagocytic system; as well as cutaneous (cutaneous leishmaniasis, CL) and mucosal forms (mucosal leishmaniasis, ML), which are characterized by involvement of skin and mucous membranes, respectively (Mcgwire and Satoskar, 2014).

The development of new therapeutic approaches for leishmaniasis treatment requires as well new high throughput screening methodologies for searching compounds with anti-leishmanial activity. Reporter gene (RG) technology has become one of the most promissory and widely used tool for drug screening in several models since it offers live imaging, high sensibility, specificity and flexibility (Pulido et al., 2012). These genes typically encode a gene product that has a readily measurable phenotype and is easily distinguishable over endogenous cellular background (Dube et al., 2009). Depending on the application, an ideal RG should be: (i) absent from the host; (ii) inert (should not affect the physiology of the parasite cell); and (iii) should represent a simple, sensitive, and inexpensive assay for quantification of reporter expression (Dube et al., 2009). The choice of a RG depends on the cell line used, the nature of the experiment, and the adaptability of the assay to the appropriate detection method (New et al., 2003).

Fluorescent RGs have been applied in the developing of new methods less laborious and more sensitive than the classical microscopy method for drug screening. The family of green fluorescent protein (GFP and multimeric gfp) was the most studied, but the main disadvantage of these RGs was the low level of fluorescence of transgenic parasites (Ha et al., 1996). Moreover, a background noise was observed, when *L. (L.) donovani* expressing the episomal GFP was used for the screening in intracellular amastigotes and promastigotes generating a problem to automatize the technique (Dube et al., 2005). Another approach was to use the enhanced GFP (egfp) trying to improve the fluorescent signals. The transfected parasites showed stable expression of the fluorescent protein and the assay provided a more accurate approach in drug sensitivity profile, but it was not automated as a high-throughput (Bolhassani et al., 2011). Then, GFP integrated into the *Leishmania* genome was evaluated, the expression was very stable and homogeneous reducing the noise generated by similar episomal genetic manipulation (Pulido et al., 2012). This allows the development of automatized methodologies with the least background. Another RG, is tandem dimer Tomato (tdTomato), a variant of DsRed, derived from the coral *Discosoma* sp, which has demonstrated photostability and a high brightness (283% of eGFP) (Shaner et al., 2004; Morris et al., 2010). TdTomato has been used successfully in transgenic mouse models, fusion protein applications, and as a promoter-reporter (Morris et al., 2010). It also demonstrated to be useful in the generation of *Trypanosoma cruzi* parasite lines that express this fluorescent protein and the use of these lines to establish accurate and simple *in vitro* as well as *in vivo* systems to screen and test anti-*T. cruzi* compounds (Canavaci et al., 2010).

On the other hand, chemotherapy remains the major control strategy for leishmaniases with meglumine antimoniate (Ma) enduring as the first-line treatment (Pan American Health Organization, 2013). However, its use in the clinical setting exhibits several limitations, given that they require an aggressive administration schedule (one or two intramuscular daily injections for 21 to 35 days), and often exhibit a wide range of local and systemic side effects. On the other hand, Pan American Health Organization (PAHO) recognizes that currently none of the drugs available for the treatment of leishmaniasis in the Americas completely eradicates the infection (PAHO, 2013). Given the above-mentioned reasons, the development of new, less toxic and more cost-effective drugs with greater efficacy as well as more accessible alternative therapeutic strategies that could become available for low-income populations to treat the disease has become a necessity (PAHO, 2013; Oryan, 2015).

In recent years, there has been growing interest in alternative natural products and plant compounds for the treatment of leishmaniases (Chouhan et al., 2014). The anti-leishmanial activity of some plant extracts has been attributed to flavonoids (Wong et al., 2014). These are a group of polyphenolic compounds that naturally present in fruits and vegetables and are known as antioxidants and anticancer with a significant protective effect against membrane damage (Sifaoui et al., 2014). Recently, we reported the activity of several catechins from *Camellia sinensis* against *L. (L.) amazonensis*, being EGCG the most active one (Sosa et al., 2020). This group of compound can form complexes with the parasite cell wall to influence processes requiring cell linking, and hence inhibit the parasite growth (Ogeto et al., 2013). In a research work carried out by Manjolin et al. (2013), it has been shown that dietary flavonoids with low cytotoxicity characteristics such as fisetin, quercetin, luteolin and 7,8-hydroxyflavone can inhibit arginase enzyme from *L. (L.) amazonensis*. Arginase plays a central role in the biosynthesis of polyamine which is very important and essential for protecting the parasite against oxidative stress and ROS produced by the host’s defense system (Oryan, 2015). Therefore, natural compounds as flavonoids are an interesting group to search potential candidate with anti-leishmanial activity.

For all the aforementioned, the aims of this work were to develop and validate a fluorescence assay based in the expression of tdTomato protein by *Leishmania (L.) amazonensis*, and to apply this method to evaluate *in vitro* the activity of flavonoids and reference drugs. In addition, we studied the mechanism of action of one active compound selected though the *in vitro* screening assay.

## Materials and Methods

### Compounds

Flavonoids: Flavonol: Galangin (GAL) – ≥95% (HPLC) (Sigma-Aldrich 282200), fisetin (FI) – P90% (HPLC) (PhytoLab PHL82542), rutin (RU) – ≥95.0% (HPLC) (PhytoLab PHL89270), morin (MO) – ≥95.0% (HPLC) (PhytoLab PHL82601). Flavanol: (-)-epigallocatechin gallate (EGCG)- >98% (HPLC) was kindly supplied by Mitsui Norin (Shizuoka, Japan).

Reference drugs: Pentamidine isethionate (PE) (Sigma-Aldrich P0547) and Miltefosine (MI) – ≥98.0% (perchloric acid titration) (Sigma-Aldrich M5571) were purchased from Sigma–Aldrich. Meglumine antimoniate (Ma) (Glucantime^®^, Aventis-Sanofi, São Paulo, Brazil) was kindly supplied by Ministerio de Salud de la Nación (Buenos Aires, Argentina).

### Parasites

Parasites of *L. (L.) amazonensis* (MHOM/BR/73/M2269) were isolated from an infected mouse. First, the isolated parasites were cultured in Difco agar containing 20% defibrinated rabbit blood (USMARU medium) plus sterile proline balanced salts solution (PBSS) with 100 U/mL penicillin and 50 µg/mL streptomycin (P-S) at 23°C. After four days, the parasites were passaged to RPMI-1640 medium (Gibco, Grand Island, NY, USA) supplemented with 10% (v/v) heat-inactivated fetal bovine serum (FBS) and P-S.

### PIR1SAT/tdTomato Plasmid Construction

The tandem dimeric tomato red fluorescent reporter gene (tdTomato, 1464 bp) was obtained by restriction enzyme digestion from the pTREX-tdTomato plasmid constructed for *Trypanosoma cruzi* (Canavaci et al., 2010). The fragment was then gel purified and cloned into the expression site of pIR1SAT plasmid (Xba and Sal1). Correct insertion of the tdTomato gene was corroborated by digestion with selected restriction enzymes. For transfection, pIR1SAT-tdTomato plasmid was linearized with SwaI enzyme, gel purified and quantified. This plasmid integrates into the *Leishmania* genome by replacing one copy of the SSU rRNA gene which is transcribed by pol1 (Buckner and Wilson, 2005). pTREX-tdTomato and pIR1SAT plasmids were kindly provided by Dr. R.L. Tarleton (University of Georgia, USA) and Dr. S. Beverley (Washington University, USA), respectively. Additional information about tdTomato and its sequence can be found at the following link https://www.fpbase.org/protein/tdtomato/.

### Transfection of *Leishmania*

Mid-log promastigotes of *L. (L.) amazonensis* were collected by centrifugation and washed once with phosphate buffer saline (PBS; pH 7.4) plus P-S. Then, parasites were resuspended in cold OPTI-MEM reduced serum medium (Gibco) at 1 x 10^8^/ml and the pIR1SAT-tdTomato (10 µg/ml) was added. The parasites and the plasmid were placed in a 2 mm gap electroporation cuvette (BTX Inc., San Diego, CA). The cuvette was chilled on ice for 10 min, then parasites were electroporated using an Electro Cell Manipulator 630 (BTX Inc.) set at 450 V, 0.025 ῼ, and 500 µF. After electroporation, the cuvette was placed on ice for 10 min, then the parasites were transferred to USMARU medium for 24 h. One-day post-electroporation, nourseothricin (NTC) (50 µg/ml) was added to select parasites expressing the streptothricin acetyltransferase (SAT) gene. Resistant parasites were cloned in a Petri dish in USMARU medium plus RPMI-1640 with 10% SFB and NTC (50 µg/ml). A diagnostic PCR was carried out in 6 clones (C2, C3, C5, C11, C12 and C21) to confirm the insertion of tdTomato gene into the *Leishmania* genome. The sequence of the primers used is detailed in **Supplementary Material**.

### Detection of Fluorescence Signal in Transfected Parasites and Its Correlation With the Number of Parasites

The fluorescence emission was measured in C2, C3, C5, C11, C12 and C21. Briefly, promastigotes in the log phase (1 x 10^6^/0.1ml) of each clone and promastigotes of the wild-type strain (*L. amaz*/wt) were plated in a 96-well black culture plate with clear bottom (Nunc, USA) and incubated at 23°C. After 24 h of incubation, the fluorescence was measured in a fluorimeter plate reader Infinite F200 (Tecan, Männedorf, Switzerland) with the optics positioned under the bottom of the plate and the filters 535/25 for excitation and 595/35 for emission. A clone (*L. amaz*/tc3) was selected for subsequent *in vitro* and *in vivo* experiments.

To determine the correlation between the fluorescence signal and the number of parasites, *L. amaz*/tc3 promastigote in the mid-log phase (3 x 10^7^, 1 x 10^7^, 3 x 10^6^, 1 x 10^6^, 3 x 10^5^, 1.2 x 10^5^, 4 x 10^4^ cells/well) and *L. amaz*/tc3 amastigotes (3 x 10^6^, 1 x 10^6^, 3 x 10^5^, 1 x 10^5^, 3 x 10^4^, 1.2 x 10^4^, 4 x 10^3^ cells/well) were plated in 0.1 ml in a 96-well black culture plate with clear bottom (Nunc, USA). In the case of amastigotes, the parasites were isolated from a macrophage culture as was described previously (Chang, 1980). Un-transfected parasites (*L. amaz*/wt) were the negative control. The fluorescence was measured in a fluorimeter plate reader (Tecan, Männedorf, Switzerland).

### *In Vitro* Assessment of Transfected Parasites Growth

In order to evaluate if the growth of *L. amaz*/tc3 was modified by the transfection with the RG, promastigotes (5 x 10^5^ p/ml) were plated in triplicate in RPMI-1640 medium in a 96-well culture plate and the positive control was *L. amaz*/wt. At the 1^st^, 3^rd^ and 7^th^ day of incubation, the density (parasites/ml) was determined using a Neubauer chamber in an optic microscope (40x) (Leitz Wetzlar, Germany).

Then, the growth of intracellular amastigotes of *L. amaz*/tc3 was evaluated. Raw 264.7 cells (5 x 10^4^/well) were cultured in complete RPMI-1640 and plated in 96-well black plate (Nunc, USA) for 3 h at 37°C in a 5% CO_2_ for macrophage adherence. After that, macrophages were infected with promastigotes of *L. amaz*/tc3 at different ratios, 1:5, 1:10 and 1:20 (macrophage:promastigotes), and incubated at 34°C in a 5% CO_2_ overnight. Infected cultures were washed with pre-warmed PBS to remove free parasites. Macrophages without infection were the negative controls. The fluorescent was measured in a plate reader (Tecan, Männedorf, Switzerland) at 1^st^, 2^nd^, 3^rd^ and 4^th^ day of culture.

### *In Vitro*, Fluorescent Assay Validation

Then, we evaluated the efficacy of MI, a reference drug for leishmaniasis by the fluorescence method (*L. amaz*/tc3) and by the conventional method based in counting the parasite at the optic microscope (*L. amaz*/wt). Mid-log promastigotes of *L. amaz*/tc3 and *L. amaz*/wt were harvest and seeded (1 x 10^6^/0.1 ml) with different concentrations of MI (40 to 0.6 µg/ml) in a 96-well plate at 23°C. Promastigotes cultured only in medium were the controls. Parasite viability was determined by measuring the fluorescence of *L. amaz*/tc3 in a plate reader (Tecan, Männedorf, Switzerland) and by counting the mobile promastigotes in a Neubauer chamber at the optic microscope (40x) (Leitz Wetzlar, Germany) at 48 h of incubation with MI. Viability was calculated with the formula: $Viability_{L.amaz/tc3\;}=\frac{FLt}{FLc}\times 100$ where, FLt is the fluorescence of treated culture, and FLc is the fluorescence of control; $Viability_{L.amaz/wt}=\frac{NPt}{NPc}\times 100$ where, NPt is the number of promastigotes in the treated culture, and NPc is the number of promastigotes in control culture. Then, the 50% inhibitory concentration (IC_50_) of MI was estimated.

Furthermore, the efficacy of MI was also determined against intracellular amastigotes of *L. amaz*/tc3 and *L. amaz*/wt. Raw 264.7 macrophages were plated in 96-well black plate (5 x 10^4^ cells/well) and in eight well Lab-Tek tissue culture slides (6.6 x 10^4^ cells/ml) (Nunc, USA). The cells were infected with promastigotes in stationary phase of *L. amaz*/tc3 and *L. amaz*/wt (1:20 ratio) at 33°C, 5% CO_2_. After incubation, the infected macrophages were washed to remove non-internalized parasites with PBS plus P-S. New RPMI-1640 medium with different concentrations of MI (20 to 0.62 µg/ml) was added to each well and incubated at 33°C, 5% CO_2_ during 48 h. The anti-leishmanial activity of MI was determined by measuring the fluorescence of intracellular amastigotes of *L. amaz*/tc3 in a plate reader (Tecan, Männedorf, Switzerland). The viability was calculated as mentioned above, and the IC_50_ was also estimated. Simultaneously, the leishmanicidal activity of MI against *L. amaz*/wt was determined by the microscopy test. In this case, the infected macrophages were stained with Diff Quik (Biopur S.R.L., Rosario, Argentina) and the number of intracellular amastigotes was determined in 200-500 macrophages in treated and control cultures per slide under the immersion lens microscope (100x) (Leitz Wetzlar, Germany). The infection index (II) and the % infectivity (I) were calculated by the following formulas: *II* = *IM* × *A*, where IM= infected macrophages (%), A= number of amastigotes per infected macrophages; and $I\left(\%\right)=\frac{IIT}{IIc}\times 100,$ where IIT= infection index in treated cultures, IIC= infection index in control cultures (Ayres et al., 2008). The IC_50_ of MI was calculated.

### Course of Infection of *L. amaz*/tc3 in Mice

Mice were bred at the mouse facility of the Faculty of Health Sciences, National University of Salta (Salta, Argentina). Female BALB/c mice (*n* = 5) of 6 weeks of age were infected in their hind right footpad with the stationary phase promastigotes (5 x 10^6^ parasites/0.05 ml) to evaluate the infectivity of *L. amaz*/tc3; animals infected with *L. amaz*/wt were the controls (*n* = 5). The footpad swelling was measured weekly with a digital caliper (150 mm; Digimess).

### Efficacy of Ma in Mice Infected With *L. amaz*/tc3

Female BALB/c mice were infected in their hind right footpad with the stationary phase of promastigotes (5 x 10^6^ parasites/0.05 ml) of *L. amaz*/tc3. Once a week the footpad swelling was measured with the digital caliper. Mice (*n* = 5) were treated with Ma in PBS (120 mg Sb/kg/day) starting at week 9 post-infection; the antimonial was administered by intraperitoneal injection (ip) once a day for 21 days. Control group (*n* = 5) received only PBS (ip). Within a week of finishing the Ma treatment, the parasite load was quantified by three methods, fluorescence (FL), optic microscope (OM) and limiting dilution assay (LD) in treated and control group. The FL method was carried out as follows, the hind right footpad was excised and weighed; then the tissue was homogenized using a glass grinder with 5 ml of complete RPMI-1640 medium and the intracellular amastigotes of *L. amaz*/tc3 were released. A sample (0.1 ml) of the homogenate was centrifuged to replace the medium for PBS, then it was plated in duplicates in a 96-well black culture plate with clear bottom (Nunc, USA) and the fluorescence was measured in a plate reader (Tecan, Männedorf, Switzerland). Besides, a calibration curve was used to extrapolate the sample fluorescence to the number of amastigotes. For that, a pool of amastigotes from control samples was used; amastigotes (1 x 10^6^ to 1.5 x 10^4^) were plated in duplicates in a 96-well black culture plate with clear bottom, and the fluorescence was measured in a plate reader (Tecan, Männedorf, Switzerland). The number of amastigotes was adjusted to the weight of the sample.

Another sample of the tissue homogenate was used to quantify the parasite loads by direct counting in a Neubauer chamber under an optical microscope (40X) (Leitz Wetzlar, Germany), and the concentration of parasites (amastigotes/ml) of each homogenate was determined (Hill et al., 1983). The total number of parasites was adjusted to the weight of each tissue sample.

Finally, a sample of the tissue homogenate was also used to quantify the parasite loads by the LD assay (Lima et al., 1997). After serial dilutions in 96-well plates, the samples were incubated at 23°C for 14 days and the number of viable promastigotes was determined from the highest dilution at which the parasite could grow.

### Drug Screening Against Promastigotes and Intracellular Amastigotes by the Fluorescence Assay

After fluorescence assay validation as it is described above, the method was used to evaluate *in vitro* the flavonoids anti-leishmanial activity. Mid-log promastigotes of *L. amaz*/tc3 were harvest and seeded with different concentrations of GAL (1,200 to 18.75 µM), MO, FI and RU (40 to 1.25 µM), and EGCG (300 to 18.75 µM). Positive control were promastigotes treated with PE (4 to 0.25 µM), the negative control were promastigotes cultured with medium only, and the blank was the culture medium. The parasites were incubated at 23°C for 48 h. The anti-leishmanial activity was determined by measuring the parasite fluorescence in a plate reader (Tecan, Männedorf, Switzerland). The viability was estimated as was described above for MI (fluorescent assay). The IC_50_ was calculated for each compound.

The flavonoids anti-leishmanial activity was also evaluated against intracellular amastigotes. Macrophages (Raw 264.7) were plated in 96-well black plate (5 x 10^4^ cell/well) and infected with promastigotes in stationary phase of *L. amaz*/tc3 (1:20 ratio) at 33°C, 5% CO_2_. After incubation, the infected macrophages were washed to remove non-internalized parasites with PBS plus P-S. New RPMI-1640 medium with different concentrations of GAL (30 to 3.75 µM), MO (300 to 37.5 µM), FI (65 to 8.12 µM), RU (300 to 37.5 µM) and EGCG (30 to 7.5 µM) was added to each well and incubated at 33°C, 5% CO_2_ for 48 h. Infected cells treated with Ma (136.6 to 34.1 µM) were the positive controls, and cells incubated only with RPMI-1640 were the negative control. The infected macrophages were incubated during 48 h at 33°C, 5% CO_2_. After treatment, the fluorescence of intracellular amastigotes of *L. amaz*/tc3 was measured in a plate reader (Tecan, Männedorf, Switzerland). The viability was determined as was described for MI (fluorescent assay). The selectivity index (SI) (CC_50_ for macrophages/IC_50_ for *Leishmania*) was calculated. A SI higher than one, was considered more selective for activity against *Leishmania*, whereas a value lower than one was considered more selective for the activity against the macrophages (Tiuman et al., 2005).

All experiments in promastigotes and intracellular amastigotes were performed in duplicate or triplicate wells for each condition and repeated at least twice.

### Drug Toxicity in a Macrophage Cell Line

The cytotoxicity against a cell line of macrophages (Raw 264.7) of GAL, MO, FI, RU, EGCG, PE, MI and MA was determined by the MTT method. Cells (3.3 x 10^5^/ml) cells were treated with different concentrations of each compound during 48 h at 37°C, 5% CO_2_. The controls were incubated with RPMI-1640 medium. Briefly, 50 µl of MTT (2 mg/mL) was added to each well and, after 4 h of incubation at 37°C, 5% CO_2_, the reaction was stopped dissolving the formazan crystals with 100 µl/well of dimethyl sulfoxide. The relative amount of formazan produced by viable macrophages was determined using a spectrophotometer at 595 nm. The percentage of viable cells was calculated according to the formula$\;\frac{\left(OD{\;}_{treated\;culture}-OD{\;}_{blank}\right)}{\left(OD{\;}_{control}-OD{\;}_{blank}\right)}\;\times 100$ as was described previously (Adinehbeigi et al., 2017). The cytotoxicity concentration 50% (CC_50_) was calculated for each compound.

### Analysis by Transmission Electron Microscopy

Ultrastructural alterations in *Leishmania* induced by flavonoids with anti-leishmanial activity were analyzed by transmission electron microscopy (TEM). Promastigotes of *L. amaz*/tc3 (1 x 10^6^ parasites/ml) were exposed to the IC_50_ of most active flavonoids selected in the drug screening. The control was a culture of *L. amaz*/tc3 incubated in complete RPMI-1640 medium. After 48 h of incubation, samples of *L. amaz*/tc3 promastigotes from each culture were fixed in modified Karnovsky’s solution (formaldehyde [2.66%], glutaraldehyde [1.66%]) and PBS 0.1M (pH 7,4), and incubated overnight at 4°C. The fixed samples were post-fixed with a 1/1 solution of PBS/2% osmium tetroxide (OsO4), overnight. Then the material was dehydrated and included in Spurr (Pelco, USA). Ultrathin sections were cut with an ultramicrotome (MT1, Sorvall, USA), mounted on copper grids, and contrasted with uranyl acetate (PELCO, USA) and lead citrate. The samples were observed with a Zeiss EM109 (Carl Zeiss, Oberkochen, Germany) transmission electron microscope, at CIME (Centro Integral de Microscopía Electrónica, CONICET-UNT-Tucumán, Argentina) and electron micrographs were taken.

### Cytokine Production by Activated Macrophages

To evaluate if the most active flavonoid against *Leishmania* can active infected macrophages to produce IL-12p70 and IL-10 cytokines, macrophages (Raw 264.7) were infected with promastigotes (ratio 1:20) of *L. amaz*/tc3 as was described previously in “*Drug Screening Against Promastigotes and Intracellular Aastigotes by The Fluorescence Assay*” section, and cultured in a 5% CO_2_ incubator. Non-infected cells (2 x 10^5^ cells/mL) were cultured in RPMI-1640 medium plus 10% FCS and 1% antibiotics (P-S) and placed into 24 well plates. Cells were exposed to 5 µg/ml of phytohaemagglutinin (PHA) (Sigma Aldrich, Missouri, USA), 5 µg/ml of concanavalin A (CONA) (Sigma Aldrich, Missouri, USA); and the most active flavonoid selected against *Leishmania* (20 µM) during 48 h. Then, the culture supernatant was recovered and maintained at -80°C until use. The culture supernatant concentrations of IL-12p70 and IL-10 (BD Biosciences, San Diego, USA) were determined by ELISA following the specifications of the supplier. Each condition was performed in quadruplicate.

### Statistical Analysis

The GraphPad Prism 5.0 software (GraphPad Software Inc., San Diego, CA, USA) was used to calculate the IC_50_ and CC_50_ values by nonlinear regression. Multiple comparisons of the parasite burden between groups were made with a one-way analysis of variance (ANOVA) followed by Tukey test. Comparisons between two groups were performed by Mann-Whitney (non-parametric t-test). The parasite loads obtained by the LD assay were estimated with the ELIDA software (Taswell, 1986). *p* < 0.05 was considered significant. For the analyses of culture supernatants by ELISA, continuous variables between two groups were compared with the Mann-Whitney U-test. A nonparametric Kruskal-Wallis test with Dunn’s post-test was used to compare differences among multiple groups.

## Results

### Generation of *L. (L.) Amazonensis* Expressing the tdTomato Protein

**Figure 1:**
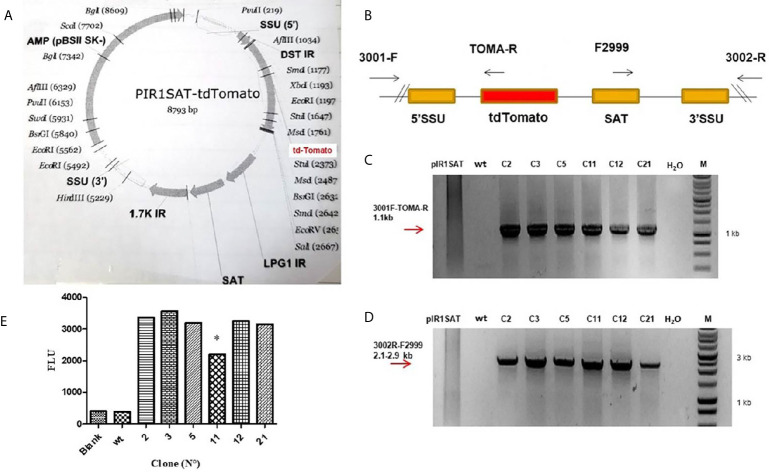
**(A)** Map of the PIR1SAT-tdTomato plasmid indicating the enzyme used for cloning and linearization for *L. (L.) amazonensis* transfection assays. **(B)** Schematic representation of expected genomic loci of transfected *Leishmania* after replacement of one copy of the SSU rRNA gene. **(C)** PCR genotyping analysis with primers 3001-F/TOMA-R confirming the expected gene insertion. **(D)** PCR genotyping analysis with primers 3002-R/F2999 also corroborating the correct insertion and replacement of SSU rRNA gene. Line 1: pIR1SAT-tdTomato plasmid, line 2: wild type parasites, lines 3-8: transfected clones, line 9: no template control (-), line 10: molecular weight and marker. Diagrams are not to scale. Numbers are sizes (kb) of expected products. **(E)** Detection of fluorescence emission in six clone selected (C2, C3, C5, C11, C12 and C21) after 24 h of incubation at 23°C (*p < 0.05). Parasite wild-type (wt). Statistical significance was assessed using ANOVA (^*^ *p* < 0.05).

The pIR1SAT-tdTomato plasmid was linearized with SwaI restriction enzyme before transfection of *L. (L.) amazonensis* parasites (**Figure 1A**). The linearized plasmid integrates into the *Leishmania* genome by replacing one copy of the SSU rRNA gene which is transcribed by pol1 (Buckner and Wilson, 2005). After the selection of transfected parasites with NTC, 6 clones out of the 23 (C2, C3, C5, C11, C12 and C21) were chosen randomly to confirm the genomic integration by PCR. The **Figure 1B** shows a schematic representation of the expected genomic loci after transfection and the primers used for PCR of selected clones. The pair of primers 3001-F and 3002-R hybridized outside the expression cassette and were used to corroborate insertion of the linearized plasmid. As we expected, PCR fragments of 1.1 kb (**Figure 1C**) and 2.1-2.9 kb (**Figure 1D** ****) were obtained when primers sets 3001-F/TOMA-R and F2999/3002-R were used respectively in the analysis of the 6 clones selected.

### Detection of Fluorescence Signal in Transfected Parasites and Its Correlation With the Number of Parasites

The fluorescence signal was detected in the six clone selected (C2, C3, C5, C11, C12 and C21) after 24 h of incubation at 23°C (**Figure 1E**). Clone 11 was the only one that showed a lower fluorescence with respect to the others (*p* < 0.05) (**Figure 1E**); this could be related with the kinetic of different clone cultures, i.e. parasites in different stages of growth could be slightly different in its metabolism, affecting the transcriptional activity of the reporter gene.

**Figure 2:**
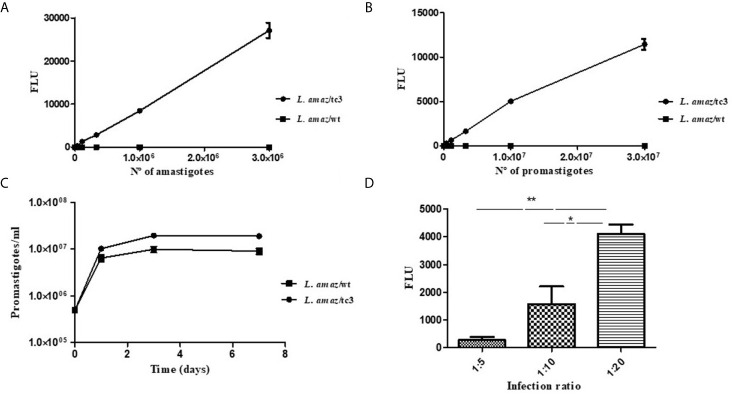
Correlation between the fluorescence signal and the number of amastigotes **(A)** and promastigotes of *L. amaz*/tc3 **(B)**. **(C)** No statistical differences were observed between the curve of growth of *L. amaz*/tc3 and *L. amaz*/wt, indicating that transfection with the RG did not modified the normal parasite growth. **(D)** The fluorescence signal of intracellular amastigotes of *L. amaz*/tc3 increased reaching the peak at the second day post-infection, and it was higher in macrophages infected with the ratio 1:20 than 1:5 and 1:10. Statistical significance was assessed using ANOVA (^*^ *p* < 0.05 and **p < 0.001).

The clone number 3 (*L. amaz*/tc3) was selected to carried out the subsequent *in vitro* and *in vivo* experiments. Promastigotes and amastigotes of *L. amaz*/tc3 showed a good correlation between the fluorescence signal and the number of parasites (*p* < 0.0001) (**Figures 2A, B**).

### *In Vitro* Assessment of Transfected Parasites Growth

On the first day of culture, the density of both, *L. amaz*/tc3 and *L. amaz*/wt, increased almost 20 times (approximately to 1 x 10^7^ p/ml). Then, from day 3 to 7, the density of fluorescent parasites and wild-type was stable showing the cultures were in the stationary phase (**Figure 2C**). No statistical differences were observed between the curve of growth of *L. amaz*/tc3 and *L. amaz*/wt, indicating that transfection with the RG did not modified the normal parasite growth.

In addition, the growth of intracellular amastigotes of *L. amaz*/tc3 was evaluated at different times and ratios of infection. The fluorescence signal increased with the infection time and reached a peak on the second day post-infection (pi) (**Figure 2D**). Besides, the fluorescence was significantly higher in macrophages infected at ratio 1:20 with respect to those infected at radio 1:5 (*p* < 0.001) and 1:10 (*p* < 0.05) (**Figure 2D**). After that, the fluorescence started to decrease in all the infected cultures (4^th^ day pi, data not shown).

### Validation of the Fluorescence Assay

Once the fluorescence was detected in promastigote and amastigote of *L. amaz*/tc3, we evaluated if the fluorescence was a good indicator of the parasite viability after treatment with an anti-leishmanial drug. For that, MI was selected since it showed activity against promastigotes and amastigotes of several species of *Leishmania*, and the efficacy of MI was determined by the fluorescent assay and by the conventional methods. After, 48 h of incubation with MI, promastigotes viability decrease, and no difference was observed between the values of IC_50_ determined by the fluorescence and microscopic test (IC_50_ = 5.25 ± 0.05 µg/ml**)**. In the same manner, MI inhibited the growth of intracellular amastigotes, and both methods showed similar results (IC_50_ = 1.65 ± 0.75 µg/ml).

### Course of Infection of *L. amaz*/tc3 in Mice

**Figure 3:**
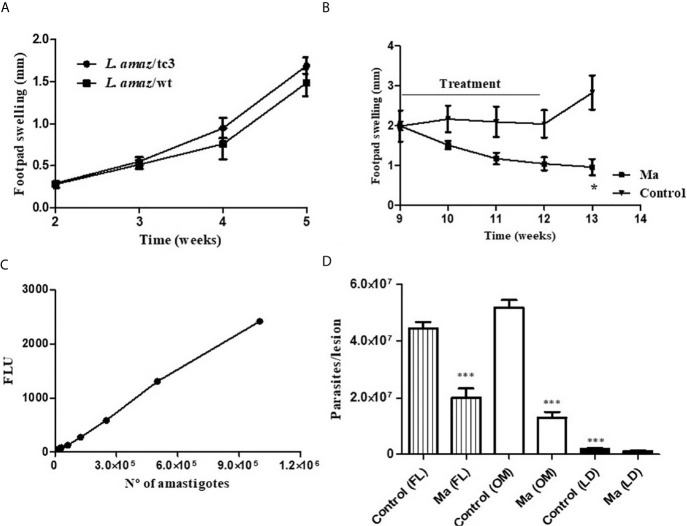
**(A)** The infectivity of *L. amaz*/tc3 in BALB/c mice was similar to the wild strain (*L. amaz*/wt). **(B)** Reduction in the footpad swelling in BALB/c mice infected with *L. amaz*/tc3 and treated with meglumine antimoniate (MA) (120 mg Sb/kg/day) (*p* < 0.05). **(C)** Calibration curve, fluorescence and number of amastigotes isolated from mice infected with *L. amaz*/tc3 (r^2^ = 0.99, *p* < 0.0001). **(D)** Parasite load quantified, at week 13 of the experiment in mice treated with Ma and control group, by fluorescence method (FL), optic microscope (OM) and limiting dilution (LD). Statistical significance was assessed using Mann-Whitney U-test t (^*^ *p* < 0.05) and ANOVA (***p < 0.001). Data (mean ± SD) are from a representative experiment (*n* = 5 mice).

The infectivity of *L. amaz*/tc3 was evaluated in BALB/c mice. After infection, the footpad swelling increased weekly as did the wild strain. At week 5 pi, no differences in the footpad swelling were observed (**Figure 3A**).

### Efficacy of Ma in Mice Infected With *L. amaz*/tc3

BALB/c mice were infected with *L. amaz*/tc3 to evaluate the efficacy of Ma. The animals were treated with the antimonial for 21 days, once a day. After one week of treatment, the footpad swelling started to decrease and it still continued after ended the treatment (week 12-13) (**Figure 3B**). At week 13 of the experiment, a significant difference in the footpad swelling was observed between Ma and the control group (*p* < 0.05) (**Figure 3B**). In addition, differences highly significant were observed in the parasite burden between Ma and control group (*p* < 0.001) for FL and OM showing 55% and 75% of inhibition, respectively. On the other hand, we observed the parasite burden determined by LD assay was significantly lower (*p* < 0.001) in both groups, Ma and control, than those quantified by FL and OM; despite this, the inhibition percentage obtained by this assay was 54%, similar to those obtained for FL (**Figures 3C, D**).

### Drug Screening Against Promastigotes and Intracellular Amastigotes by the Fluorescence Assay

First, the anti-leishmanial activity of a group of flavonols (GAL, MO, FI and RU) was assessed in promastigotes cultures. Also, we compared the activity of this group with EGCG, a catechin flavanol with leishmanicidal activity that we reported previously (Sosa et al., 2020). GAL showed activity on *L. amaz*/tc3 and the IC_50_ value was 2.2 fold lower (53.09 ± 16.56 µM) than EGCG (119.8 ± 17.6 µM); while no inhibition in the parasite growth was observed for MO, FI and RU with doses ≤ 40 µM. The reference drug PE, used as the positive control, was highly active against promastigotes (IC_50_ = 0.67 ± 0.18 µM) after 48 h of incubation.

Then, we evaluated the efficacy of flavonols (GAL, MO, FI and RU), EGCG and the reference drug (Ma) in the macrophage-amastigote model by the fluorescent assay. After treatment, the fluorescence was measured in the plate reader. The results caught our attention, since no relationship between the drug concentrations and the fluorescence was observed in GAL, MO and FI. The background fluorescence increased with concentrations ≥ 30 µM (GAL and FI) or ≥ 75 µM (MO), and it could neither be subtracted from the blank nor be eliminated by washing the cell culture. It is important to mention, the auto-fluorescence of macrophage cells and culture medium was very low compared to the fluorescence of td-tomato (data not shown). Therefore, we could not determine the IC_50_ for GAL, MO and FI by the fluorescent assay; instead, we estimated the percentage of amastigotes inhibition in those concentrations where the background did not interfere the fluorescence readout. GAL and FI showed a similar percentage of inhibition on the parasites (29-31%) with 15 µM **(** **Table 1** **)**. On the other hand, MO showed a low inhibition (13.5%) against amastigotes at 37.5 µM. In the case of RU and Ma, not background fluorescence was detected, and the IC_50_ values were 171 µM and 80.31 µM, respectively **(** **Table 1** **)**.

**Table 1:** *In vitro* anti-leishmanial activity and cytotoxicity of flavonoids and the reference drug, MA.

METHOD		GAL	MO	RU	FI	EGCG	Ma
**Microscopic**	**IC_50_ μM (SE)**	**20.59 (± 4.47)**	**122.4 (± 2.55)**	**133 (± 8.25)**	**> 60**	**130 (± 10.01)**	**ND**
**Fluorescence**	**IC_50_ μM (SE)**	**ND**	**ND**	**171**	**ND**	**ND**	**80.31 (± 14.7)**
	**Inhibition (%)**	**29**	**13.5**	**ND**	**31**	**20**	**ND**
**Colorimetric**	**CC_50_ μM (SE)**	**22.75 (± 2.87)**	**> 230.2**	**> 240**	**52.57 (± 6.14)**	**193.9 (± 23.8)**	**> 1,000**
	**SI**	**1.1**	**> 1.8**	**> 1.8**	**< 1**	**1.5**	**> 12.5**

ND, Not determined; SE, Standard error; SI, Selectivity index = CC_50_ for macrophages/IC_50_ for Leishmania.

Then, to estimate the IC_50_ values of GAL, FI and MO; and to confirm the IC_50_ of RU, the activity was evaluated quantifying the intracellular amastigotes at the optic microscope. We followed the same methodology described for MI (microscopic assay) in “*In Vitro, Fluorescent Assay Validation*” section and the range of concentrations were for GAL (30 to 7.5 µM), FI (60 to 15 µM), RU and MO (200 to 50 µM), EGCG (142 a 17.7 µM). The IC_50_ of GAL (20.59 ± 4.47 µM) was 5.9 and 6.4 times lower than the IC_50_ of MO (122.4 ± 2.55 µM) and RU (133 ± 8.25 µM), respectively. Furthermore, GAL was more active than the flavanol EGCG (IC_50_ = 130 ± 10.01 µM) **(** **Table 1** **)**.

Referred to the toxicity, GAL (SI = 1.1), MO (SI > 1.8), RU (SI > 1.8), EGCG (SI = 1.5) and Ma (SI > 12.5) were more selective against the parasites than the host cells with SI values higher than 1. Instead, FI was more selective against the host cells than the parasite (SI < 1) **(** **Table 1** **)**.

### GAL and EGCG Induced Ultrastructural Alterations in *Leishmania*

**Figure 4:**
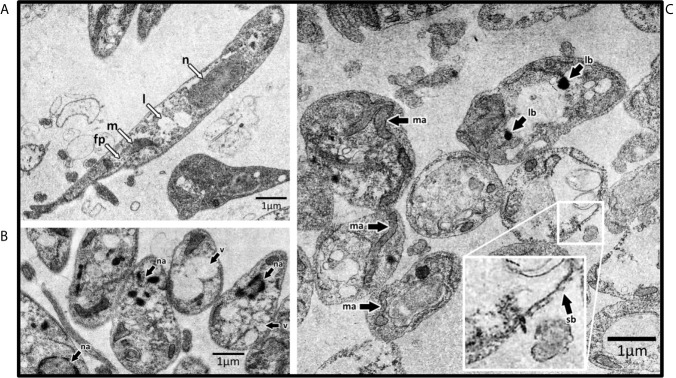
Transmission electron microscopy of *L. amaz*/tc3 promastigotes. **(A)** Untreated culture with normal cellular structures: nucleus (n), lipid inclusion (l), mitochondrion (m), and flagellar pocket (fp). Black arrows indicate ultrastructural alterations in parasites exposed to EGCG **(B)** and GAL **(C)** nuclear alterations (na), vacuolization (v), alterations in mitochondrial structure (ma), lipid-storage bodies (lb), and surface blebbing (sb).

Through the *in vitro* drug screening, we found GAL was the most active compound against *Leishmania*. Thus, we investigated the effects of this compound on the ultrastructure of *L. amaz*/tc3 promastigotes by transmission electron microscopy (TEM). In addition, we analyzed the effects of EGCG, another compound that also showed anti-leishmanial activity. As controls we used untreated *L. amaz*/tc3 promastigotes. As we expected, control promastigotes presented a typical elongated morphology with normal cellular structures such as nucleus, lipid inclusion, mitochondrion, and flagellar pocket **(** **Figure 4A** **)**. Both GAL and EGCG induced different alterations in the ultrastructure of promastigotes, showing intense cellular damage **(** **Figures 4B, C** **)**. GAL produced dramatic changes in the mitochondrial structure, with the formation of enlarged and swollen mitochondria compared to the untreated control, cytoplasmic lipid bodies as well an increase in plasma membrane blebs (surface blebbing) **(** **Figure 4C** **)**. EGCG induced intense cytoplasmic vacuolization and also the presence of nuclear membrane detachment, chromatin condensation, and marginalization **(** **Figure 4B** **)**. Moreover, the morphology of the parasites exposed to the drugs became visibly altered, with loss of most of the cytoplasmic organelles and a predominant round shape, confirming cellular death.

### GAL Induced the Production of Th1 Cytokine in Infected Cultured Macrophages

**Figure 5:**
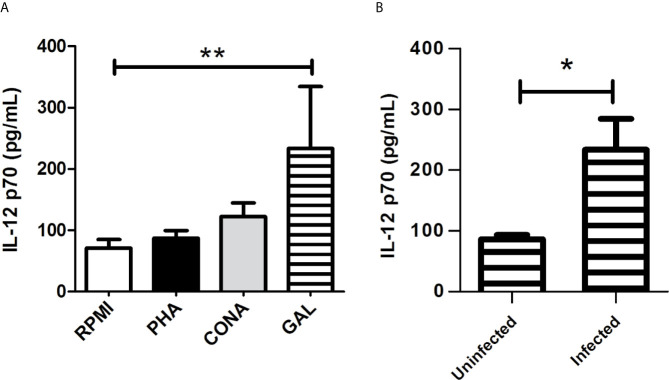
Culture supernatant production of IL-12 p70 by *Leishmania* infected macrophages treated with GAL. **(A)** The amount of IL-12p70 was assessed in infected cultured RAW 264.7 macrophages maintained alone (RPMI) or treated with phytohemagglutinin (PHA), concanavalin A (CONA) and galangin (GAL). Each variable was performed in quadruplicate. Comparison among groups were assessed by Kruskal-Wallis test and Dunn`s post-test (**p < 0.01). **(B)** Production of IL-12p70 after the addition of GAL to uninfected *vs*. infected cultured macrophages. Analysis performed by Mann Whitney test (*p < 0.05).

We determined in the culture supernatant the concentration of two cytokines produced by activated macrophages: IL-12 p70, required for the induction of IFN-γ production; and IL-10, which is known to diminish the production of inflammatory mediators. We compared the production of IL-12 p70 and IL-10 between the different culture treated cells: infected and non-infected macrophages alone (RPMI medium) or exposed to PHA, CONA, and GAL. GAL was evaluated in this assay since this compound was the most active against *Leishmania*. Infected cells treated with GAL showed higher production of IL-12 p70 [mean (range) 232.2 pg/ml (137.5-331.7 pg/ml)] compared to infected non-stimulated controls (RPMI medium) [72.11 pg/ml (56.38-84.20 pg/ml)] (*p* < 0.01) **(** **Figure 5A** **)**. Moreover, the addition of GAL to infected macrophages induced a significant increase of IL-12 p70 in comparison to non-infected macrophages treated with the same stimulus (*p* < 0.05) **(** **Figure 5B** **)**. IL-10 concentration did not show significant differences between the different treated groups (data not shown).

## Discussion

A search for new chemotherapeutic compounds for leishmaniasis diseases is still a challenge as well as the development of new methods to evaluate potential anti-leishmanial drugs. Moreover, the emergence of drug resistance and the treatment failure to antimonial make this search imperative (Agnew et al., 2001; García-Bustos et al., 2021).

In this sense, we obtained a clone of *L. (L.) amazonensis* (*L. amaz*/tc3) expressing the tdTomato. Its tandem dimer structure plays an important role in the exceptional brightness of this protein (283% of eGFP and 160% of DsRed) (Shaner et al., 2004). It is important to mention, that tdTomato gene was integrated into *Leishmania* genome by replacing a copy of SSU rRNA gene; this region has a high expression level since it is transcribed by polymerase 1, and it is highly conserved among *Leishmania* species (Buckner and Wilson, 2005).

As was expected, promastigotes and amastigotes of *L. amaz*/tc3 showed a strong fluorescence signal, and it was correlated with the number of parasites. On the other hand, the genetic manipulation of parasites did not alter either, the *in vitro* growth, or the infectivity in BALB/c mice showing similar behavior to the wild-type strain (*L. amaz*/wt).

*In vitro*, the fluorescence assay was validated with MI since promastigote are not sensitive to MA (Robert et al., 1995). MI showed a similar efficacy against both *L. amaz*/tc3 and the wild type (*L. amaz*/wt), indicating that transfection and the constitutive tdTomato expression did not affect the anti-leishmanial activity of the drug. The IC_50_ values determined for MI by the fluorescence assay in our study was consistent with the published data that demonstrated an effective dose of 1.31 μg/ml and 7.54 μg/ml for amastigotes and promastigotes of *L. (L.) amazonensis*, respectively (Escobar et al., 2002; Morais-Teixeira et al., 2011).

Then, the fluorescence assay was validated *in vivo*. BALB/c mice were infected with *L. amaz*/tc3 and *L. amaz*/wt and treated with Ma, the drug efficacy was determined by fluorescence and LD assay. Both methods showed a similar percentage of inhibition in the parasite load (54-55%) at the end of treatment, but the fluorescence assay was more sensitivity than the LD. The sensitivity difference observed between the methods might be because LD assay only detect amastigotes capable of transforming into promastigotes and surviving in the medium culture; instead, the fluorescence assay quantifies all the amastigotes present in the crude extract of lesion macerate able of expressing the tdTomato protein. Besides, the parasite load determined by the fluorescent assay was consistent with the optic microscope indicating the amastigotes were viable. In agreement with our results, the luminescence assay in *ex vivo* tissue also showed a higher sensitivity than the LD (Reimão et al., 2013).

The fluorescence assay developed in this work, through the expression of tdTomato RG, was a simple system for drug screening. This method, unlike those based on catalytic reporter genes (β-lactamase, luciferase, β-galactosidase) (Okuno et al., 2003; Ashutosh et al., 2005; Buckner and Wilson, 2005), has the advantage that not require an addition of reagent, and the fluorescence readout can be done at different times of incubation. Besides, we highlight the stability of the fluorescence of *L. amaz*/tc3. We observed that transfected parasites showed fluorescence 6 months’ post-infection in BALB/c mice (data not shown). The fluorescence stability in infected animals is important in studies that require a long period of evaluation like relapses of treatments or in vaccines studies. Furthermore, these transgenic parasites can be used at *in-vivo* experiments taking advantage of the tdTomato emission range, which is easily detected at 620 nm, outside of the range of most of the animal tissue auto-fluorescence (Canavaci et al., 2010).

Several fluorescent proteins, applied in drug screening assay, have been studied during the last three decades. The GFP was the most studied, but the main disadvantage of this RG was the low level of fluorescence of transgenic parasites (Ha et al., 1996). Then, trying to improve the fluorescent signals, egfp was studied. The transfected parasites showed stable expression of the fluorescent protein and the assay provided a more accurate approach in drug sensitivity profile, but it was not automated as a high-throughput (Bolhassani et al., 2011). On the contrary, our system was automatized using a plate reader, to measure the fluorescence in promastigotes drug screening and in amastigotes isolated from treated animals.

Through the fluorescence assay, we selected the flavonol GAL as the most active compound against promastigotes of *L. amaz*/tc3. Another work reported the activity of GAL isolated from *Alpinia galanga* rhizomes against promastigotes of *L. (L.) donovani*, the causal agent of visceral leishmaniasis, but the parasite inhibition was reached at 100 µM (IC_50_), (Kaur et al., 2010) almost twice the IC_50_ in *L. amaz*/tc3 determined in this work. The differences in the sensitivity might be related to the species of *Leishmania* as it was showed by Escobar et al. (2002).

It is worth to mention that in the macrophage-amastigote model, we observed an interference in the tdTomato fluorescence readout, and it increased with drug concentrations of GAL, MO and FI. This interference was not associated with the auto-fluorescence of macrophages and culture medium because they were very low compared to the tdTomato. Mukai et al. (2009) demonstrated that flavonol aglycones like GAL, MO, isorhamnetin and quercetin, presented auto-fluorescence (ex 488 nm and em 515–535 nm) in Hepa-1c1c7 cells treated with these compounds; moreover, it was concentration-dependent. They suggested the auto-fluorescence might be related with the hydroxyl group at the C3-position in the flavonol skeleton since no fluorescence was observed in the cells treated with compounds belonging to the flavone, flavanone and catechin subclasses. Therefore, we hypothesized the interference in the tdTomato readout might be due to an overlap between the extremes of the emission spectra of tdTomato and green fluorescence of the flavonol aglycones when they are excited at 535 nm. On the other hand, we did not observe interference in the tdTomato fluorescence readout when infected macrophages were treated with RU and Ma and we could determine the IC_50_ of these compound. The activity of GAL, MO, FI and EGCG was determined by optic microscopy.

After all, GAL was the most active compound against promastigotes and amastigotes of *L. amaz*/tc3. GAL produced morphological alterations on mitochondria and plasma membrane of promastigotes of *L. amaz*/tc3 with loss of the cytoplasmic organelles. Moreover, the ability of GAL to induce IL-12p70 up-regulation by *Leishmania* infected macrophages *in vitro* indicates that this natural compound could play an important role in the eradication of the invading parasites. Optimal production of both fractions of IL-12 (p40 and p70) is required to drive a predominant T helper 1 response with the subsequent IFN-γ production, macrophage activation and NO- mediating effects needed to kill the pathogens (Mattner et al., 1996; Guler et al., 2011).

All these data demonstrates that the fluorescence assay, based on the stable expression of the tdTomato protein by *L. (L.) amazonensis*, is a useful tool to assess the anti-leishmanial activity of new drugs. However, some interference in the tdTomato readout can occur in the macrophage-amastigote model when flavonol aglycones are been testing. Further studies are necessary to identify if there are others natural compounds that might affect the performance of the fluorescence assay. Besides, the present study demonstrated the potential inhibitory effect of GAL against *L. (L.) amazonensis* and its capacity to induce the production of IL-12 p70, a cytokine related to a Th1 response (cure); therefore, these results encourage us to study its efficacy in animal model in near future.

## Data Availability Statement

The original contributions presented in the study are included in the article/**Supplementary Material**. Further inquiries can be directed to the corresponding author.

## Ethics Statement

The animal study was reviewed and approved by Animal Ethics Committee of the Faculty of Health Sciences, National University of Salta, Salta, Argentina (Resolution N° 309–18).

## Author Contributions

MG-B, PB, CPB, AM, CP, JM, AS, VB, OP, PM, and PP performed experiments. MG-B, PB, CPB, CP, and JM analyzed data. MG-B and PB designed experiments. MG-B and PB drafted the manuscript. All authors contributed to the article and approved the submitted version.

## Funding

This work was funded by Agencia Nacional de Promoción Científica y Tecnológica and by GLAXO, PICT-O 2011-0017 (PB), by the Research Council of the Catholic University of Salta, Argentina (Grant Resolution 1494/16) and by the Research Council of the National University of Salta (Type B Project No. 2411) (MG-B).

## Conflict of Interest

The authors declare that the research was conducted in the absence of any commercial or financial relationships that could be construed as a potential conflict of interest.
