# Supplementary material for: Development of a Fluorescent Assay to Search New Drugs Using Stable tdTomato-Leishmania, and the Selection of Galangin as a Candidate With Anti-Leishmanial Activity
Source: Front Cell Infect Microbiol. 2021 Jun 4;11:666746. doi: 10.3389/fcimb.2021.666746 (PMC8213385; doi:10.3389/fcimb.2021.666746)
Supplement: Supplementary file 1 [file DataSheet_1.pdf]

Development of a fluorescent assay to search new drugs using stable tdTomato-*Leishmania*, and the selection of galangin as a candidate with anti-leishmanial activity

María Fernanda García-Bustos[1,2,3], Agustín Moya Álvarez[2], Cecilia Pérez Brandan[2], Cecilia Parodi[2], Andrea Mabel Sosa[2], Valeria Carolina Buttazzoni Zuñiga[1], Oscar Marcelo Pastrana[1], Paula Manghera[1], Pablo Alejandro Peñalva[1], Jorge Diego Marco[2], Paola Andrea Barroso[2]*

Supplementary Material

Set of primers to confirm by PCR the integration of tdTomato into the *Leishmania* genome

Primer F2999 5’-CCTAGTATGAAGATTTCGGTGATC-3’

Primer 3001-F 5’-GATCTGGTTGATTCTGCCAGTAG-3’

Primer 3002-R 5’-CTGCAGGTTCACCTACAGCTAC-3’

Primer TOMA-R 5'- GCTCACCCTAGGCGCCAT- 3'
